# Development of a Complex Intervention for Effective Management of Type 2 Diabetes in a Developing Country

**DOI:** 10.3390/jcm11051149

**Published:** 2022-02-22

**Authors:** Tigestu Alemu Desse, Kevin Mc Namara, Helen Yifter, Elizabeth Manias

**Affiliations:** 1 School of Nursing and Midwifery, Centre for Quality and Patient Safety Research, Institute for Health Transformation, Faculty of Health, Deakin University, Geelong, VIC 3217, Australia; emanias@deakin.edu.au; 2 Deakin Rural Health, School of Medicine, Faculty of Health, Deakin University, Geelong, VIC 3217, Australia; kevin.mcnamara@deakin.edu.au; 3 Deakin Health Economics, Institute for Healthcare Transformation, Deakin University, Geelong, VIC 3217, Australia; 4 Department of Internal Medicine, College of Health Sciences, Addis Ababa University, Addis Ababa 9086, Ethiopia; helenefbr@gmail.com

**Keywords:** type 2 diabetes, complex intervention, behaviour change intervention, co-design, continuity of care, developing country, Ethiopia, patient participation, patient transfer

## Abstract

There has been little focus on designing tailored diabetes management strategies in developing countries. The aim of this study is to develop a theory-driven, tailored and context-specific complex intervention for the effective management of type 2 diabetes at a tertiary care setting of a developing country. We conducted interviews and focus groups with patients, health professionals, and policymakers and undertook thematic analysis to identify gaps in diabetes management. The results of our previously completed systematic review informed data collection. We used the United Kingdom Medical Research Council framework to guide the development of the intervention. Results comprised 48 interviews, two focus groups with 11 participants and three co-design panels with 24 participants. We identified a lack of structured type 2 diabetes education, counselling, and collaborative care of type 2 diabetes. Through triangulation of the evidence obtained from data collection, we developed an intervention called VICKY (patient-centred collaborative care and structured diabetes education and counselling) for effective management of type 2 diabetes. VICKY comprised five components: (1) patient-centred collaborative care; (2) referral system for patients across transitions of care between different health professionals of the diabetes care team; (3) tools for the provision of collaborative care and documentation of care; (4) diabetes education and counselling by trained diabetes educators; and (5) contextualised diabetes education curriculum, educational materials, and documentation tools for diabetes education and counselling. Implementation of the intervention may help to promote evidence-based, patient-centred, and contextualised diabetes care for improved patient outcomes in a developing country.

## 1. Introduction

Type 2 diabetes is a global public health problem and an economic burden to nations, particularly developing countries [1]. It contributes to cardiovascular complications, such as ischemic heart disease, heart failure, and renal disorders [2,3,4].

Ineffective management of type 2 diabetes has been associated with poor clinical outcomes, which include disease progression, and increased health services utilisation, such as repeated hospitalisations and high all-cause mortality [5,6,7]. In Sub-Saharan Africa (SSA) [8,9,10] including Ethiopia [11,12,13], there exists a high rate of diabetes-related morbidity and mortality, high cost of diabetes care, and poor quality of life for patients with type 2 diabetes. Excessive levels of diabetes-related problems and high cost of type 2 diabetes care in SSA are attributed to widespread lack of treatment success, stemming from inadequate organisational involvement and delivery of care [8,9,14]. Multiple contributing factors exist in the region, such as a lack of contextually tailored diabetes management approaches, inadequate diabetes training of health professionals, and low levels of collaborative care and effective shared treatment plans developed by patients and health professionals [10,14,15,16]. In Ethiopia, similar factors contributing to ineffective management of type 2 diabetes exist, including inadequate collaborative care among pharmacists, physicians, and nurses; lack of structured diabetes education; and high levels of medication therapy problems and diabetes complications [13,17,18,19,20,21,22,23]. 

Patient-centred collaborative care and the use of culturally tailored interventions, including behavioural interventions, can improve diabetes care in low-income countries [14,24,25]. Evidence indicates that SSA nations require evidence-based type 2 diabetes management strategies tailored to the context and aimed at reducing diabetes-related morbidity and mortality and high healthcare costs [10,16,26]. However, there has been little focus on designing contextually tailored type 2 diabetes management strategies in this region. While evidence suggests that structured diabetes education and counselling and collaborative care by pharmacists, physicians, nurses, and other health professionals can improve health outcomes and cost of type 2 diabetes treatment [27,28,29,30], implementation needs for such elements of care are not readily understood for type 2 diabetes in Ethiopia [21,22,31,32]. Furthermore, studies examining type 2 diabetes in Ethiopia are mainly observational and focused on the rate of glycemic control, magnitude of diabetes-related complications, quality of care, and mortality. Moreover, there has been no focus on designing appropriately tailored interventions to improve diabetes care [12,13,18,33,34,35,36,37,38]. To the authors’ knowledge, there has been no pragmatic study undertaken to explore the dynamics of current management for type 2 diabetes at a micro- or meso-level or devise much-needed diabetes management strategies tailored to SSA [10,14,16,39,40]. 

The aim of this study was to develop a theory-driven, tailored, and context-specific complex intervention for the effective management of type 2 diabetes at a tertiary care setting of a developing country.

## 2. Materials and Methods

The study was undertaken at the diabetes centre of a tertiary teaching hospital (Tikur Anbessa Specialised Hospital) in Addis Ababa, Ethiopia. Diabetes care is provided at the diabetes centre of the hospital by endocrinologists, endocrinology fellows, internal medicine residents, and nurses [41]. Each month, the diabetes centre serves about 1200 ambulatory patients with type 2 diabetes [42]. 

**Figure 1:**
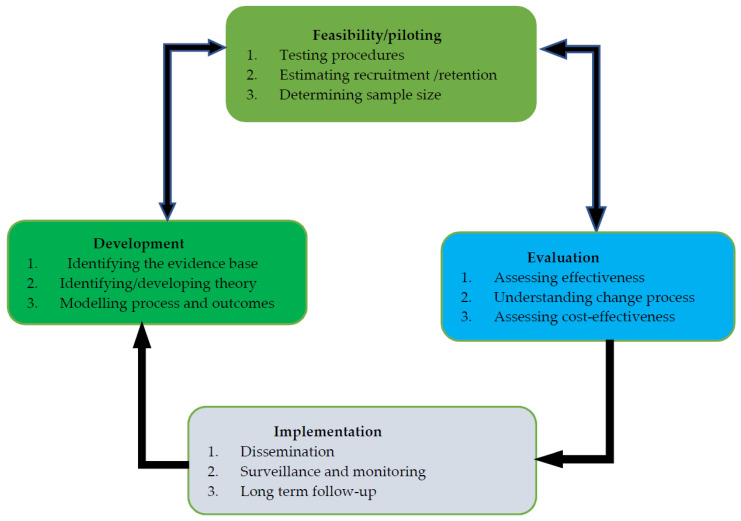
Key elements of the development and evaluation process (Craig et al., 2008). Reproduced with permission of the UK Medical Research Council.

The United Kingdom Medical Research Council (UK MRC) [43] framework was used to guide the development of a complex intervention. The MRC framework comprises detailed information about the systematic development of interventions. It utilises the best available evidence and appropriate theory to develop an intervention using a carefully phased approach [43]. The framework has four key elements (Figure 1) [43]: developing a complex intervention, feasibility and piloting, evaluation, and implementation. 

### 2.1. Developing a Complex Intervention

Developing a complex intervention involves three steps (Figure 1): (1) identifying the evidence base, (2) identifying and developing an appropriate theory of the intervention, and (3) modelling the process and outcomes of a complex intervention [43]. This study used all three steps throughout the development of the intervention. 

### 2.2. Identifying the Evidence Base

The first stage in the development of a complex intervention is to identify an existing, relevant evidence base [43]. We undertook a systematic review on the effectiveness of clinical pharmacy interventions on health and economic outcomes of patients with type 2 diabetes [30]. We also completed semi-structured interviews and focus groups with adult patients with type 2 diabetes, health professionals, and policymakers of Tikur Anbessa Specialised Hospital (TASH) and the Ministry of Health of Ethiopia to generate evidence and identify gaps in the management of type 2 diabetes at the hospital. We brought all the relevant evidence obtained through the systematic review [30], interviews, and focus groups together to understand the issues relating to effective and ineffective management of type 2 diabetes and the relevant behaviours that could be targeted for the intervention.

### 2.3. Identifying and Developing Theory

Identification and development of appropriate theory in intervention design is key to understanding the possible processes of change [43,44]. The use of a theoretical approach in the design of healthcare interventions has been demonstrated to improve the effectiveness of the interventions [44,45,46]. In this study, the Behaviour Change Wheel (BCW) framework [46,47] was used as a guide to develop an evidence-based behaviour change intervention for the effective management of type 2 diabetes. Use of the BCW supplements the MRC framework to design effective complex interventions to change behaviour in a healthcare system [44,48]. The framework can be used to develop interventions at any level (individuals, groups, and organisations) in healthcare systems [47]. The BCW framework has been effectively implemented in developing behaviour change interventions in healthcare [49,50,51,52,53]. The theory of the complex intervention for this study focused on designing an organisational level intervention, as this approach has been demonstrated to improve the effectiveness of type 2 diabetes management in previous studies [54,55,56]. 

**Figure 2:**
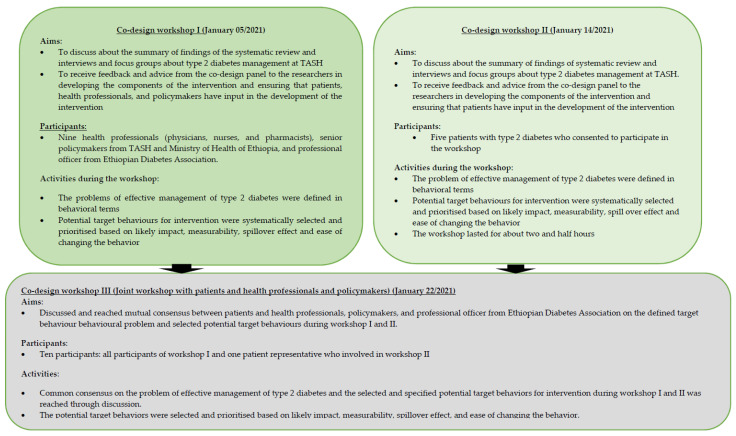
Activities undertaken in the co-design workshops.

We used a co-design panel comprising patients, health professionals, and policymakers at TASH and the Ministry of Health of Ethiopia with a representative of the Ethiopian Diabetes Association and incorporated the findings from the systematic review [30], interviews, and focus groups to inform the initial stages of development of the theory of the complex intervention. We conducted three consecutive co-design workshops with the co-design panel to help with the first two stages of the BCW [47] (Figure 2). 

#### 2.3.1. Workshop I

The first co-design workshop involved health professionals, policymakers from TASH and the Ministry of Health of Ethiopia, and a professional officer from the Ethiopian Diabetes Association (Figure 2). During the workshop, we sought to define the problems affecting the effective management of type 2 diabetes in behavioural terms and selected potential target behaviours deemed to improve the management of type 2 diabetes. The co-design panel also discussed the findings of interviews and focus groups and validated that they truly reflected the existing challenges of diabetes care at the diabetes centre of TASH. The panel then defined the problem of suboptimal management of type 2 diabetes in behavioural terms and identified potential target behaviours for the intervention that would help to improve the management of type 2 at TASH using the findings from the systematic review [30], interviews, and focus groups [42]. The identification of potential target behaviour based on impact, measurability, changeability, and spillover effect was undertaken by rating each list of potential target behaviours identified via interviews and focus groups as unacceptable, unpromising but worth considering, promising, and very promising, by each participant of the co-design panel [47]. 

#### 2.3.2. Workshop II

We conducted the second co-design workshop with patients with type 2 diabetes (Figure 2). The purpose of undertaking workshop II was to incorporate the views and experiences of patients and engage them in the intervention design. In this workshop, patients discussed the findings of the systematic review [30], interviews and focus groups, and validated these findings; they defined the problem related to facilitating effective management of type 2 diabetes in behavioural terms; and selected potential target behaviours deemed to address the problem. In this workshop, each co-design panel member rated and identified potential target behaviours as described for workshop I. The panel also elected and assigned one patient amongst the group who participated in the third co-design workshop. 

#### 2.3.3. Workshop III

A joint workshop was undertaken with a nominated patient, health professionals, policymakers, and a professional officer from the Ethiopian Diabetes Association. The workshop involved examining the defined problem related to the effective management of type 2 diabetes and the selected potential target behaviours during the two separate workshops (workshop I and II). The workshop panel members specified the target behaviours that were agreed upon. In workshop III, the panel members discussed and reached a consensus on the defined problem related to effective management of type 2 diabetes and the selected potential target behaviours at workshops I and II (Figure 2). The criteria for prioritisation and selection of the potential target behaviours for the intervention in workshop III followed the same procedures used in workshops I and III. The co-design panel in the third workshop specified the potential target behaviours for the intervention in terms of the following:

- Who needs to perform the behaviour?
- What do they need to do differently to achieve the desired change?
- When do they need to do it?
- Where do they need to do it?
- How often do they need to do it?
- With whom do they need to do it?

**Figure 3:**
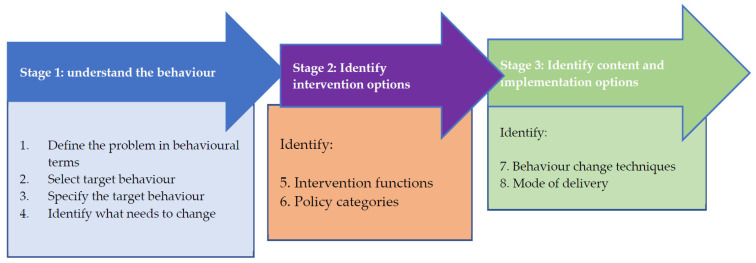
Behaviour change intervention design process (Michie et al., 2014).

The co-design panel worked through stage one to stage three of the behaviour change intervention design process [47]. The steps in the BCW (Figure 3) were sequentially explored by the co-design panel throughout the three workshops, to both ensure that the appropriate behaviours were targeted, and the intervention functions were achievable and practical in the context of TASH.

##### Stage One

This stage involved four steps (Figure 3) [47]. Step I involved defining the problem in behavioural terms (i.e., being specific about the target individual, group, or population involved in the behaviour and the behaviour itself). Step II comprised selecting the target behaviour for the intervention among a list of behaviours [47]. Step III involved specifying the target behaviour. Step IV comprised identifying what needs to change for the behaviour to change in terms of capability, opportunity, and/or motivation in the target population, group, or individual [47].

##### Stage Two 

This stage involves the use of the behavioural diagnosis [47] to:

- Decide what ‘intervention functions’ to apply: education, persuasion, incentivisation, coercion, training, restriction, environmental restructuring, modelling, and enablement;
- Select implementation strategy: fiscal policy, legislation, regulation, environmental planning, communications, service provision, and guidelines development.

##### Stage Three

The focus of the third stage is to:

- Develop a detailed intervention plan by selecting from among a range of specific behaviour change techniques (BCTs) [57]. Michie identified 93 BCTs within 16 groupings. We used Michie’s BCTs [57] to characterise components for the behavioural intervention in this study;
- Create the detailed intervention specification covering all aspects of content and delivery of the intervention structured around the chosen BCTs and modes of delivery.

Appropriate intervention functions, BCTs, and intervention contents were determined through discussion between the co-design panel and the research team and using the APEASE criteria [47]. The APEASE criteria refer to affordability, practicability, effectiveness, acceptability, safety/side effects, and equity [47]. 

### 2.4. Modelling and Creating a Complex Intervention

Modelling of a complex intervention [43] helps to precisely describe and comprehend the interaction of individual intervention components, and perceive possible effects of the intervention [58]. The careful design of a model of a complex intervention is a critical step in designing tailored and contextualised interventions in healthcare systems and choosing appropriate outcomes so that the benefits and risks of the interventions are demonstrated effectively [58].

In this study, we operationalised the intervention functions and BCTs into a complex intervention to improve the effectiveness of type 2 diabetes management. The researchers collaborated with the co-design panels in operationalising the intervention functions and BCTs into the mode of care delivery using the BCW framework [47]. We used the Revised Standards for Quality Improvement Reporting Excellence: (SQUIRE 2.0) publication guidelines [59] to report the findings of this study (Supplementary Materials Table S1).

## 3. Results

### 3.1. Study Participants 

We undertook interviews with 48 participants and two focus groups (n = 11) with patients with type 2 diabetes, health professionals, and policymakers from TASH and the Ministry of Health of Ethiopia [42] comprising an overall sample of 59 participants; three co-design workshops (n = 24); and a systematic review on the effectiveness of clinical pharmacy interventions on health and economic outcome of patients with type 2 diabetes [30] to help with the intervention design.

### 3.2. Step One: Define the Health Problem in Behavioural Terms 

Evidence from the interviews and focus groups we have undertaken, previous findings [11,12,13,60], feedback from the co-design workshops, and the context of the hospital enabled identification of the health problem. We identified that improving the effectiveness of type 2 diabetes management for patients with type 2 diabetes was the specific problem existing at the diabetes centre of TASH. 

### 3.3. Step Two: Select the Target Behaviour

We identified through interviews, focus groups, and co-design workshops that had challenges for the effective management of type 2 diabetes related to:

1. Lack of resources, such as medications, laboratory, and diagnostic tests;
2. Lack of continuity of care, such as prolonged follow up clinic visits;
3. Lack of knowledge and awareness of patients about type 2 diabetes and its complications;
4. Lack of self-care activities;
5. Low level of type 2 diabetes education and counselling services;
6. Low competence and experience of health professionals providing diabetes care;
7. Inefficient collaboration among health professionals (nurses, physicians, and pharmacists) in the care of type 2 diabetes;
8. Absence of involvement of clinical pharmacists, dietitians or nutritionists, and psychologists in the care of type 2 diabetes.

Our findings from interviews and focus groups demonstrated that the problem of the effective management of type 2 diabetes can be addressed through multiple behaviours targeted in a complex intervention. These include: ensuring continuity of care; enabling provision of structured type 2 diabetes education and counselling by competent health professionals; providing collaborative care of type 2 diabetes, involving clinical pharmacists, dietitians or nutritionists, and psychologists in type 2 diabetes care; improving health professionals’ competency, commitment and professional ethics; and improving the referral system of patients with type 2 diabetes between TASH and other health institutions [42]. The co-design panels in workshop I and II discussed the identified list of potential target behaviours for the intervention that helped with improving the effective management of type 2 diabetes at TASH. 

During the co-design workshop, the co-design panel prioritised the potential target behaviours, out of which the four potential target behaviours are listed from highest to lowest priority:

1. Provide structured diabetes education and counselling with competent health professionals;
2. Enable collaborative care of type 2 diabetes;
3. Involve clinical pharmacists, dietitians or nutritionists, and psychologists in the care of type 2 diabetes as members of the collaborative care team;
4. Improve health professionals’ competency, commitment, and professional ethics through trainings.

Similarly, the co-design panel in workshop II identified and prioritised the following potential target behaviours for intervention in descending order of priority.

1. Ensure continuous availability of medications;
2. Ensure continuous availability of laboratory and diagnostic tests;
3. Involve clinical pharmacists, dietitians or nutritionists, and psychologists in the care of type 2 diabetes as members of the collaborative care team;
4. Enable collaborative care of type 2 diabetes;
5. Integrate all type 2 diabetes care services at the diabetes centre.

Given the evidence from the interviews, focus groups, and previous findings [22,30,61], based on the context of the hospital, and the “less is more approach” of the BCW [47], it was beneficial to start the intervention with few behaviours and build upon these incrementally [47]. The panels in the co-design workshop III then identified and agreed that the effective management of type 2 diabetes at TASH may most likely be improved through the provision of structured diabetes education, counselling, and collaborative care (involving clinical pharmacists, dietitians or nutritionists, and psychologists) of type 2 diabetes. The panels agreed that these behaviours could easily be changed, measured, and be shared by other health professionals and health facilities with the available resources. 

The co-design panels confirmed that there was no involvement of clinical pharmacists, dietitians or nutritionists, and psychologists in the provision of type 2 diabetes care. It was found that there was a profound deficiency of the collaborative care of type 2 diabetes at the diabetes centre of TASH. A collaboratively working care team is more likely to be responsive, efficient, and provide improved care [61]. As multiple behaviours interact and play a role in the provision of structured diabetes education and counselling and collaborative care of type 2 diabetes [62,63,64,65], the co-design panel and the research team targeted changing the behaviours of the health professionals (physicians, nurses, and pharmacists, and dietitians or nutritionists) to improve the care of type 2 diabetes at TASH.

### 3.4. Step Three: Specify the Target Behaviour

After the selection of the potential target behaviours for intervention, the co-design panel in workshop III specified the two target behaviours, namely to enable the provision of structured type 2 diabetes education, counselling, and collaborative care of type 2 diabetes. These details are found in the table of Supplementary Materials (Table S2).

The findings of the interviews, focus groups, and co-design panel workshops indicated the need for the involvement of physicians, nurses, clinical pharmacists, dietitians or nutritionists, psychologists, and peer diabetes educators in the provision of structured diabetes education and counselling with patients or family members (caregivers) to improve the care of type 2 diabetes at TASH. The structured diabetes education involved the education of patients with type 2 diabetes about the condition, its complications, and management and self-care activities (Table S2).

In enabling the collaborative care of type 2 diabetes, physicians, nurses, clinical pharmacists, dietitians or nutritionists, and psychologists would work in coordination with patients and their families (caregivers), administrative bodies of the hospital, and the Ministry of Health of Ethiopia. A collaborative care team would be organised at the diabetes centre of TASH. The duties and activities of each member of the diabetes care team are described in the table of Supplementary Materials (Table S2).

### 3.5. Step Four: Identify What Needs to Change 

We used the COM-B system [47] to identify health professionals’ and policymakers’ capabilities (C), opportunities (O), and motivations (M) for providing or not providing structured diabetes education, counselling, and collaborative care of type 2 diabetes (Table S3). The research team performed behavioural diagnosis through triangulation of the findings of the interviews, focus groups, the systematic review [30]; and feedback from the co-design panels and the research team discussions. This information was used to determine what needed to change to enable health professionals to provide structured type 2 diabetes education, counselling, and collaborative care of type 2 diabetes at TASH.

#### 3.5.1. Structured Type 2 Diabetes Education and Counselling

The provision of structured diabetes education and counselling at TASH was hampered by a lack of availability and involvement of trained and qualified multidisciplinary health professionals in diabetes education and counselling (C). Insufficient time for the consultation of patients (O) and inadequate space (O) led to a lack of physical opportunity to provide structured diabetes education and counselling about type 2 diabetes. Patient adherence to diabetes educations sessions (O) negatively affected the provision of type 2 diabetes education at TASH. A triangulation of evidence from the interviews, focus groups, systematic review [30], co-design workshops, and the research team discussions and behavioural analysis ensured that there is a need to change the psychological capability, physical and social opportunity, and reflective and automatic motivation of health professionals to achieve the provision of structured type 2 diabetes education and counselling of type 2 diabetes at TASH (Table S3).

#### 3.5.2. Collaborative Care 

Time shortages and inappropriate space (O), poor communication among health professionals (O), lack of commitment and motivation of health professionals and policymakers (M), and absence of policies and guidelines for collaboration (O) contributed to a lack of collaborative care of type 2 diabetes at TASH. We triangulated the findings from interviews, focus groups, the co-design panel workshops, and the research team discussions and performed the behavioural analysis using the COM-B [47]. We analysed that the psychological capability, physical and social opportunity, and reflective and automatic motivation of health professionals have to be changed in order to provide collaborative care of type 2 diabetes at TASH (Table S3). 

### 3.6. Step Five: Identify Intervention Functions

Intervention functions appropriate to the context of TASH and that help to improve the management of type 2 diabetes were determined using the APEASE criteria [47] (Table S4).

#### 3.6.1. Intervention Functions for the COM-B Components of the Target Behaviour Provision of Structured Diabetes Education and Counselling

We used the BCW [47] mapping matrix to link the identified COM-B components; namely, psychological capability, physical and social opportunity, automatic and reflective motivation for the target behaviour, and provision of structured diabetes education and counselling with intervention functions. Based on the results of the APEASE criteria [47], we identified five intervention functions (Table S4); namely, education, training, environmental restructuring, modelling, and enablement that help with the intervention to bring about change in the targeted behaviour [47]. 

#### 3.6.2. Intervention Functions for the COM-B Components of The Target Behaviour in Collaborative Care of Type 2 Diabetes

We linked the COM-B components of the collaborative care of type 2 diabetes (psychological capability, physical and social opportunity, and automatic and reflective motivation) with intervention functions using the BCW [47] mapping matrix to identify intervention functions for the collaborative care of type 2 diabetes. We identified that education, incentivisation, training, environmental restructuring, modelling, and enablement were the most appropriate and pertinent intervention functions to the existing context of TASH in helping to change the target behaviour (collaborative care) (Table S4).

### 3.7. Step Six: Identifying Policy Categories for the Target Behaviours’ Provision of Structured Diabetes Education, Counselling, and Collaborative Care of Type 2 Diabetes

After identification of the intervention functions, we evaluated the appropriate policy categories that support the delivery of the intervention functions using the APEASE criteria [47]. Guidelines, environmental/social planning, and service provision were deemed appropriate to our context to support the intervention functions for the target behaviour provision of structured diabetes education and counselling. To support the delivery of the intervention functions for the target behaviour provision of the collaborative care of type 2 diabetes, guidelines, regulation, environmental/social planning, and service provision were the policy categories identified that were deemed appropriate to our context (Table S4).

### 3.8. Step Seven: Identifying Behaviour Change Techniques

Behaviour change techniques are active components of an intervention designed to change behaviour [57] that help to characterise the active components of the healthcare intervention [66]. We specified BCTs deemed to be the most effective and feasible in our context of improving the provision of structured diabetes education, counselling, and collaborative care of type 2 diabetes through the triangulation of a literature review; findings of the interviews, focus groups, and the systematic review [30]; and using the APEASE criteria [47]. We linked the intervention functions identified in step five with the most commonly used BCTs described in the Behaviour Change Technique Taxonomy version 1 (BCTTv1) [57] and identified the following 12 BCTs for the target behaviour provision of structured diabetes education and counselling (Table S5):

1. Feedback on behaviour;
2. Self-monitoring of behaviour;
3. Prompt/cues;
4. Salience of consequences;
5. Instruction on how to perform the behaviour;
6. Demonstration of the behaviour;
7. Restructuring the physical environment;
8. Restructuring the social environment;
9. Adding objects to the environment;
10. Goal setting behaviour;
11. Action planning;
12. Social support (unspecified).

### 3.9. Step Eight: Mode of Delivery and Development of the Complex Intervention 

**Figure 4:**
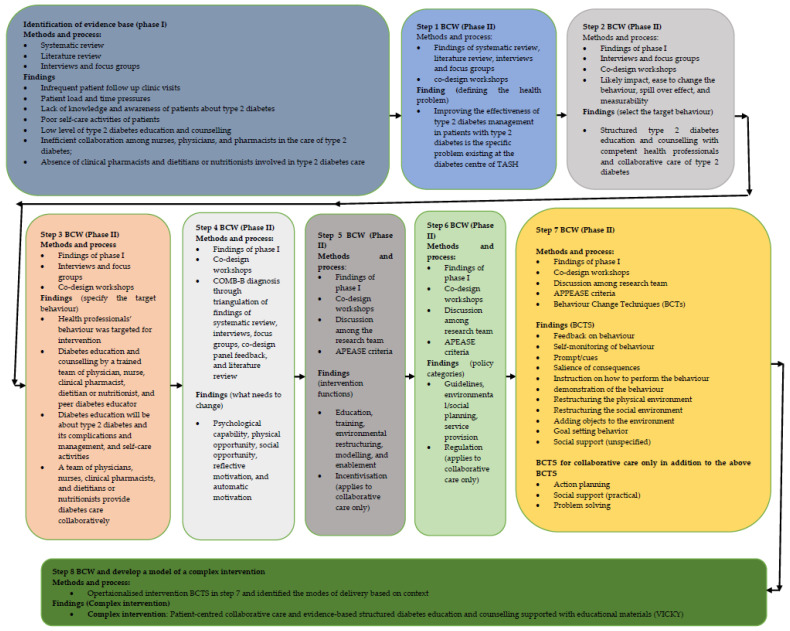
The processes involved in modelling and creating the complex intervention.

We operationalised the identified BCTs and identified modes of delivery for the provision of structured education and counselling with trained diabetes educators and collaborative care of type 2 diabetes and developed a complex intervention (Table S6). We created a complex intervention called VICKY (Patient-centred collaborative care and evidence-based structured diabetes education and counselling supported with educational materials) to improve the management of type 2 diabetes at TASH. Figure 4 summarises the development of the complex intervention according to the first stage of the UK MRC framework [43].

The complex intervention (VICKY) consisted of five components (Table 1).

The following 13 BCTs were linked to the intervention functions for the target behaviour in the collaborative care of type 2 diabetes (Table S5).

1. Self-monitoring of behaviour;
2. Prompt/cues;
3. Feedback on behaviour;
4. Instruction on how to perform the behaviour;
5. Restructuring the physical environment;
6. Restructuring the social environment;
7. Adding objects to the environment;
8. Demonstration of the behaviour;
9. Goal setting behaviour;
10. Action planning;
11. Social support (unspecified);
12. Social support (practical);
13. Problem solving.

**Figure 5:**
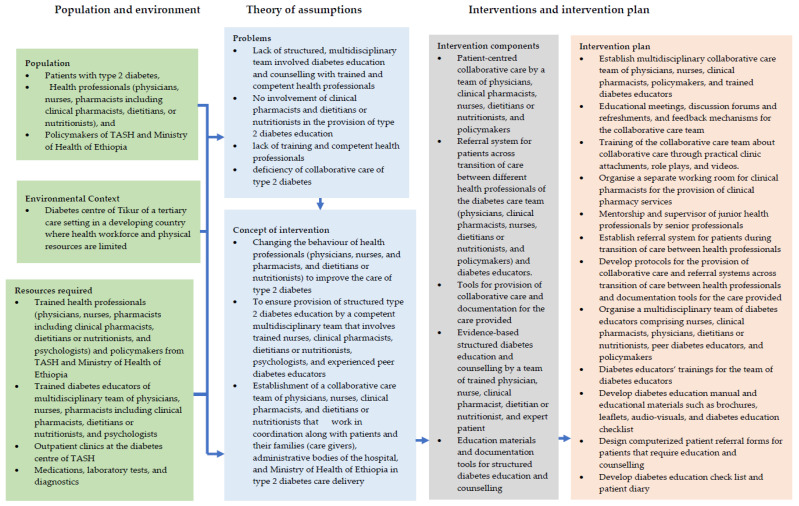
Logic Model linking context of the healthcare system, resources, and intervention activities (Conrad et al., 1999).

We used the logic model (Figure 5) to link the context of the healthcare system, such as study setting, the resources, intervention activities, theory, and assumptions underlying the intervention, and the intervention plan, in a logical order [67,68].

## 4. Discussion

This paper describes a systematic development of a tailored complex intervention to improve the effectiveness of the management of type 2 diabetes in a tertiary care setting of a developing country. To our knowledge, the complex intervention is the first theory-driven and context-specific intervention designed using the first stage of the UK MRC framework and the BCW and co-design approaches for the management of type 2 diabetes in Ethiopia.

Our intervention addresses an organisational level intervention that involves multiple stakeholders and multifaceted approaches, such as the training of health professionals, provision of educational materials, collaborative care, and patient involvement in the care process. Multifaceted approaches have been demonstrated to be successful in improving healthcare in resource-limited settings, including SSA [69,70,71,72]. Moreover, multi-level involvement comprising patient and healthcare provider-targeted interventions are likely to be successful in improving healthcare [73,74,75].

Implementation science offers opportunities to design novel healthcare approaches to ensure the utilisation of resources for evidence-based healthcare delivery in developing countries, including SSA [76,77]. Efforts have also been undertaken to enhance the use of implementation science in SSA [76,78,79] in view of the feasibility and effectiveness of implementation science in the healthcare intervention in this setting [70,76,80]. The resources available for healthcare in SSA are limited, which therefore requires the design, testing, and implementation of novel approaches for healthcare [81,82]. In this study, a novel approach for diabetes care that is based on the context of the available resources of a tertiary care setting in a developing country [22,31,32,41,83,84,85], has been designed. The intervention developed in this study may be of value in improving the quality and outcomes of diabetes care at the study setting and to tailor similar diabetes care strategies in other healthcare settings in the country [84,86]. The evidence also indicated that healthcare implementation strategies in low-income countries, such as SSA, would be feasible, sustainable, and of interest to policymakers if they are designed based on the contexts of the settings in these countries [71,76,80].

The MRC framework [43] guided the identification of an evidence base, development of theory, and modelling processes and outcomes. The BCW [47] was used to develop a theory-driven intervention, identify intervention strategies, and create elements of the complex intervention tailored to the context of the setting. We used the BCW, as it is a comprehensive framework that considers the context in intervention design [47]. Theory-driven interventions designed for patients with diabetes have been demonstrated to improve care delivery and patient outcomes [44]. Complex interventions are likely to work best if tailored to local contexts [43]. A systematic review of behavioural interventions to improve glycemic control in patients with diabetes indicated that tailored behavioural interventions improved glycemic control of patients with type 2 diabetes [87].

There have been tailored complex interventions [44,48] designed using the UK MRC [43] framework and the BCW [47] for diabetes care in developed countries. Previously developed interventions [44,48] lacked the triangulation of multiple data sources, such as interviews, focus groups, and a co-design approach in their intervention design. The distinguishing feature of our intervention design is the use of multifaceted data sources, such as consumers, health professionals of various disciplines and key policymakers, a literature review, and systematic review [30], and extensive feedback from the co-design panels comprising individuals of diverse backgrounds in contextualising the intervention. Our intervention addresses a tailored and evidence-based strategy in diabetes care delivery in a resource-limited setting.

We designed an organisational level intervention to improve the effectiveness of type 2 diabetes management. There is a broad range of evidence internationally in support of organisational interventions to improve the care of type 2 diabetes and patient outcomes [54,55,56]. Similarly, health system interventions that involved patient-centred collaborative care with multiple health professionals and diabetes education have shown effectiveness in improving the glycemic control of patients with type 2 diabetes in both developing and developed countries [56,88,89]. As multiple behaviours interact and play a role in the provision of structured diabetes education, counselling, and collaborative care of type 2 diabetes [75,76,77,78], the intervention was targeted at changing the health professionals’ behaviour involved in diabetes care delivery. Similar interventions that targeted changing health professional behaviour were found to be effective in improving diabetes care and patient outcomes [44,48,90,91,92]. In a systematic review of behaviour change interventions, such as education, training, collaborative care including physicians, nurses, and pharmacists, audit and feedback targeted at health professionals were effective in improving healthcare delivery and patient outcomes [93]. Successful management and the improved outcome of diabetes requires interaction and implementation of multiple behaviours of different health professionals, such as motivation and commitment, diabetes management knowledge and skills, interprofessional or intraprofessional communications, and compassion [62,63,64,65,91,94]. As a result, modifying multiple behaviours of professionals of various disciplines helps to improve the management of diabetes and patient outcomes [62,63,64,65,94].

Diabetes care models of developed countries are evidence-based, patient-centred, team-based, and guided by contextually tailored diabetes management guidelines and educational materials, where diabetes education by trained diabetes educators are essential elements of care [95,96,97]. Studies demonstrated a lack of collaborative care of diabetes, diabetes training of health professionals, diabetes guidelines, and diabetes education in SSA [8,9,10,98], including in Ethiopia [22]. This situation is partly attributed to the lack of facility-specific evidence about contextual factors, such as the socio-economic factors of diabetes care in SSA and the failure to tailor the diabetes care approach to the context of the SSA setting [10,42]. Therefore, it is essential to understand the specific context of a SSA diabetes care setting and design an evidence-based diabetes care strategy tailored to the context of this setting [9,99].

Our proposed intervention involves the collaborative care of diabetes, diabetes training of health professionals, and diabetes education by a trained team of health professionals. Evidence also indicates that the diabetes care team needs to incorporate a multidisciplinary group involving physicians, nurses, clinical pharmacists, dietitians or nutritionists, and psychologists [62,63,64,65]. Expanding diabetes management to several healthcare team disciplines helps patients receive the most optimal and cost-effective diabetes care and achieve better treatment outcomes [65,100,101]. Structured diabetes education is also a key component of the intervention in this study, which has been previously demonstrated to improve diabetes care delivery and treatment outcomes [30,102,103,104,105]. The evidence also indicates that multicomponent educational interventions significantly improved the glycemic control of patients with type 2 diabetes [106]. In general, our intervention, which focuses on collaborative care and structured diabetes education, will fill the gaps identified in diabetes management in SSA in general, and Ethiopia in particular [8,9,10,22,98].

This study has some limitations. We did not include the views of nutritionists or dietitians, psychologists, and laboratory personnel in the intervention design. The intervention was designed at a single healthcare setting, requiring feasibility and piloting prior to evaluation and implementation. Nevertheless, the information obtained can be transferred to other similar settings.

## 5. Conclusions

This paper indicated the usability and applicability of the UK MRC framework and the BCW to designing tailored and evidence-informed behaviour change interventions in SSA. We developed the UK MRC-guided intervention called patient-centred collaborative care and structured diabetes education and counselling (VICKY) using the BCW. VICKY, which is a tailored intervention to the context of a tertiary care setting of a developing country, is a complex intervention for diabetes management to be tested for feasibility and effectiveness in later phases of this project. This intervention will help to manage diabetes effectively by addressing the current practice gap existing at the hospital, and the country in general. VICKY is a comprehensive diabetes care model co-designed by key stakeholders involving consumers, healthcare providers of various disciplines, and policymakers using multiple evidence sources. This model, if found effective, may serve as a springboard to design similar tailored interventions for other non-communicable diseases in the country.

## Figures and Tables

**Table 1:** Components and intervention plan of the complex (VICKY) intervention.

Components of the Complex Intervention (VICKY)		Intervention Plan and Activities
1	Patient-centred collaborative care by a team of physicians, clinical pharmacists, nurses, dietitians or nutritionists, psychologists, and policymakers.	Establish a multidisciplinary collaborative care team of physicians, nurses, clinical pharmacists, dietitians or nutritionists, psychologists, policymakers, and trained diabetes educators Educational meetings, refresher trainings, discussion forums and refreshments, and feedback mechanisms for the collaborative care team Training of the collaborative care team about collaborative care through practical clinic attachments, role plays, and videos. Organise a separate working room for clinical pharmacists for the provision of clinical pharmacy services. Mentorship and supervision of junior health professionals by senior professionals. Establish a referral system for patients during transition of care between health professionals.
2	Referral system for patients across transition of care between different health professionals of the diabetes care team (physicians, clinical pharmacists, nurses, dietitians or nutritionists, psychologists, and policymakers).	
3	Tools for provision of collaborative care and documentation for the care provided.	Protocol that guides the diabetes care team for the provision of collaborative care and referral systems across transition of care between health professionals. Develop checklists to document the services provided by the collaborative care team to ensure collaborative care was provided Checklists and documentation tools such as clinical pharmacy services documentation forms that support the provision of collaborative care activities.
4	Evidence-based structured diabetes education and counselling by a team of trained physician, nurse, clinical pharmacist, dietitian or nutritionist, and expert patient.	A multidisciplinary team of individuals comprising nurses, clinical pharmacists, physicians, dietitians or nutritionists, peer diabetes educators, and policymakers will be established as a team of diabetes educators at the diabetes centre of TASH. Diabetes educators’ training tailored to the context of the hospital and the country will be provided to the multidisciplinary team of nurses, clinical pharmacists, physicians, dietitians or nutritionists, peer diabetes educators, and policymakers to produce trained diabetes educators at the diabetes centre. Context-specific diabetes education manual and educational materials such as brochures, leaflets, audio-visuals. Design computerised patient referral forms for patients that require diabetes education and counselling. Contextualised diabetes education checklist and patient diary will be developed.
5	Educational materials and documentation tools for structured diabetes education and counselling.	

## Data Availability

The data presented in this study are available on request from the corresponding author.

## Supplementary Materials

The following supporting information can be downloaded at: https://www.mdpi.com/article/10.3390/jcm11051149/s1, Table S1: Revised Standards for Quality Improvement Reporting Excellence: (SQUIRE 2.0) publication guidelines, Table S2: Behavioral specification to improve the management of type 2 diabetes at the diabetes centre of a tertiary teaching hospital, Table S3: COM-B diagnosis using the Behaviour Change Wheel for the identified target behaviours, Table S4: Linking intervention functions to COM-B components using the Behaviour Change Wheel linking Matrix, Table S5: COM-B diagnosis, intervention functions, policy categories, and behaviour change techniques for the identified target behaviours for intervention, and Table S6: Mode of delivery structured diabetes education.
