# Supplementary material for: Development of a Complex Intervention for Effective Management of Type 2 Diabetes in a Developing Country
Source: J Clin Med. 2022 Feb 22;11(5):1149. doi: 10.3390/jcm11051149 (PMC8911192; doi:10.3390/jcm11051149)
Supplement: Supplementary file 1 [file jcm-11-01149-s001.zip › jcm-1589678-supplementary.pdf]

Table S1: Revised Standards for QUality Improvement Reporting Excellence : S(SQUIRE 2.0) publication guidelines

**Text section and item name**	**Page/line no(s).**
	**info is located**
**Title and abstract**	
1. **Title**	1
Indicate that the manuscript concerns an initiative to improve healthcare (broadly defined to include the quality, safety, effectiveness, patient-centredness, timeliness, cost, efficiency and equity of healthcare).	
2. **Abstract**	2
a. Provide adequate information to aid in searching and indexing.	
b. Summarise all key information from various sections of the text using the abstract format of the intended publication or a structured summary such as: background, local problem, methods, interventions, results, conclusions.	
**Introduction: Why did you start?**	5&6
3. **Problem description** - Nature and significance of the local problem.	5&6
4. **Available knowledge** - Summary of what is currently known about the problem, including relevant previous studies.	5&6
5. **Rationale** - Informal or formal frameworks, models, concepts and/or theories used to explain the problem, any reasons or assumptions that were used to develop the intervention(s) and reasons why the intervention(s) was expected to work	5&6
6. **Specific aims** - Purpose of the project and of this report.	6
**Methods: What did you do?**	

7. **Context** - Contextual elements considered important at the outset of introducing the intervention(s).	6

8. **Intervention(s)**	
a. Description of the intervention(s) in sufficient detail that others could reproduce it.	
b. Specifics of the team involved in the work.	9-12
9. **Study of the intervention(s)**	9-12
a. Approach chosen for assessing the impact of the intervention(s).	
b. Approach used to establish whether the observed outcomes were due to the intervention(s).	N/A
10. **Measures**	
a. Measures chosen for studying processes and outcomes of the intervention(s), including rationale for choosing them, their operational definitions and their validity and reliability.	N/A
b. Description of the approach to the ongoing assessment of contextual elements that contributed to the success, failure, efficiency and cost.	N.A
c. Methods employed for assessing completeness and accuracy of data.	
11. **Analysis**	
a. Qualitative and quantitative methods used to draw inferences from the data.	9-12
b. Methods for understanding variation within the data, including the effects of time as a variable.	N/A
12. **Ethical considerations** - Ethical aspects of implementing and studying the intervention(s) and how they were addressed, including, but not limited to, formal ethics review and potential conflict(s) of interest.	13
**Results: What did you find?**	
13. **Results**	13
a. Initial steps of the intervention(s) and their evolution over time (eg, time-line diagram, flow chart or table), including modifications made to the intervention during the project.	16-23
b. Details of the process measures and outcomes.	16-23

c. Contextual elements that interacted with the intervention(s).	16-23
d. Observed associations between outcomes, interventions and relevant contextual elements.	N/A
e. Unintended consequences such as unexpected benefits, problems, failures or costs associated with the intervention(s).	N/A
f. Details about missing data.	N/A
**Discussion: What does it mean?**	
14. **Summary**	
a. Key findings, including relevance to the rationale and specific aims.	24
b. Particular strengths of the project.	24-27
15. **Interpretation**	
a. Nature of the association between the intervention(s) and the outcomes.	N/A
b. Comparison of results with findings from other publications.	24-27
c. Impact of the project on people and systems.	24-27
d. Reasons for any differences between observed and anticipated outcomes, including the influence of context.	24-27
e. Costs and strategic trade-offs, including opportunity costs.	N/A
16. **Limitations**	
a. Limits to the generalizability of the work.	28
b. Factors that might have limited internal validity such as confounding, bias or imprecision in the design, methods, measurement or analysis.	28
c. Efforts made to minimise and adjust for limitations.	28
**Conclusions**	

a. Usefulness of the work.	29
b. Sustainability.	29
c. Potential for spread to other contexts.	29
d. Implications for practice and for further study in the field.	29
e. Suggested next steps.	29
**Other information**	
18. **Funding** - Sources of funding that supported this work. Role, if any, of the funding organisation in the design, implementation, interpretation and reporting.	30

Ogrinc G, et al. BMJ Qual Saf 2015;0:1–7. doi:10.1136/bmjqs-2015-004411
Downloaded from http://qualitysafety.bmj.com/ on January 2, 2017

Table S2: Behavioral specification to improve management of type 2 diabetes at diabetes centre of a tertiary teaching hospital

Target behaviour relevant to improving type 2 diabetes management at the diabetes centre of TASH	Who	What	Instruments	Where	When	How often	With whom
Provision of structured diabetes education with trained and competent health professionals	• Physicians in charge of type 2 diabetes care, nurses, clinical pharmacists, dietitians or nutritionists, and trained peer diabetes	• Provide structured type 2 diabetes education and counselling to patients with competent health professionals about:<br>o diabetes self-management education and support (DSMES)<br>o self-care activities that include healthy eating, being active, diabetes monitoring, taking medications and medication adherence and reminders to adherence, problem-solving skills such as self-awareness and management of hyper-/hypo-glycemia, reducing the risk of complications, and healthy coping<br>o diabetes and its complications, the burden of diabetes, treatments (including modern and traditional treatments), and treatment goals for type 2 diabetes.<br>o insulin, insulin storage and administration techniques, and dose adjustment of insulin<br>o improve knowledge and awareness of patients about self-care (healthy eating, physical exercise, blood glucose monitoring, regular follow up, medication adherence and reminders to adherence, diabetes foot care),<br>• Provide continuing medical education (CME) and professional development (CPD) training to health professionals involved in type 2 diabetes care and treatment about	Use different diabetes education techniques<br>• Brochures, pamphlets, and bulletins<br>• Recorded audio-visuals<br>• Trainers, guidelines for training	At the patient waiting area of the diabetes centre	In the morning, from 8 am to 8:40 am at every visit of the patient to the hospital, CME and CPD training will be provided at	In the morning from 8am to 8:40 am at every visit of the patient at the hospital and whenever the patient needs and enquires	Patients and their families (care givers)

	educators	ethical and professional type 2 diabetes care and education, teaching and learning methods, and behaviour change theories or models to enhance their competence in diabetes care and education.<br>• Improve health professionals' diabetes education and communication skills<br>• Train physicians involved in type 2 diabetes care about medications intensification to reduce physician inertia. The training also involves improving the commitment and punctuality of health professionals, including compassionate and respectful care of type 2 diabetes<br>• Assign personnel responsible for the organisation and administration of structured diabetes education services<br>• Organise a multidisciplinary diabetes education team that includes trained peer diabetes educators and advisory committee			times untrained staff is assigned at the diabetes centre and updates on diabetes care and its management is required,	help over the phone. Frequency of training to health professionals is to be determined based on the need	
Collaborative care of type 2 diabetes	Physicians, nurses, clinical pharmacists, and dietitians or nutritionists.	• Establish a multidisciplinary collaborative care team that involves physicians, nurses, clinical pharmacists, policymakers, and trained diabetes educators for type 2 diabetes care and develop the detailed role, accountabilities, and responsibilities for each member of the team<br>• Assign coordinator for collaboration and establish mechanisms to improve communication among health professionals such as regular meetings of the multidisciplinary type 2 diabetes care team and provide refreshment services at regular meetings to enhance communication and friendly relationships among the	Protocol for collaboration containing clear goals, rules & regulations for collaborative care of type 2 diabetes and	At the diabetes centre of TASH and A separate	At patient diagnosis, initiation of treatment, and	At certain intervals of time, the multidisciplinary team, the	Patients and their families (caregivers), administrative

		health professionals, patients, and policymakers, establish feedback mechanism among the care team, organise seminar and journal club sessions about type 2 diabetes.<br>• Mentorship & supervision to improve awareness of health professionals by policymakers (administrative bodies of the hospital & ministry of health) about collaboration.<br>• Patients involve in decision making on the management of their diabetes and work towards the improvement of their diabetes<br>• The diabetes educators team provides diabetes education<br>• Patient referral to the respective collaborative care team member (health professional or diabetes educator) where special need patient support by a specific health professional is required.<br>• Assign dedicated clinical pharmacists at the diabetes centre as the type 2 diabetes management multidisciplinary team members.<br>• The clinical pharmacists provide the following services for type 2 diabetes:<br>o Patient medication review and discussions with physicians on drug therapy and making recommendations on treatment modifications, starting insulin therapy and prescribing extra amounts of insulin; titrate or adjust insulin doses (upon the Doctor's approval) when necessary.<br>o Follow patients closely to guide them through insulin dose adjustment based on their blood glucose records, develop individualised pharmaceutical care plans, implement the plans, and monitor and review them.<br>o Evaluation of antidiabetic and related medication regimens and dose up-titration per pre-established protocols; evaluation of patients' therapeutic	the roles and duties of each member of the team and a separate room for clinical pharmacists for the provision of clinical pharmacy services	room was assigne d for the clinical pharma cist at the diabetes centre of TASH.	during follow up.	coordinat or, and policyma kers may decide. Clinical pharmaci sts are involved in diabetes care at every patient visit on the type 2 diabetes clinic days.	bodies of the hospital , and Ministr y of Health of Ethiopi a,

		regimens based on efficacy, safety, adverse effects, drug interactions, drug costs, and monitoring and recommending to physicians.<br>o Consultation of other health care teams on pharmacotherapy; discuss and titrate medication regimen based on previously formulated medication titration algorithms for blood pressure, cholesterol, glycemic control and adjust doses of medications according to hepatic and renal functions.<br>o Communicating regularly with patients and their caregivers, interim contact with intervention patients via telephone or in-person to determine whether medication changes were implemented, and address questions or concerns since the previous encounter (e.g., side effects, adverse events, or adherence issues).<br>o Monitoring and follow up of complications and the patients.<br>o Clinical pharmacists provide education about type 2 diabetes and its complications, treatment and goals of treatment, about lifestyle changes (healthy diet, physical activity), provide printed educational materials such as bulletins, pamphlets, and blood glucose monitoring diary, about medications and their side effects, adverse reactions, storage, and administration especially for insulin, hypoglycemia, medication adherence, the importance of self-monitoring of blood glucose, and adjustment of insulin dosage; education on management of hyperglycemia and hypoglycemia.<br>o Organise seminar sessions and journal clubs about medications for health professionals working at the diabetes centre.					

Table S3: COM-B diagnosis using the Behaviour Change Wheel for the identified target behaviours

**Target behaviour**	**Provision of structured diabetes education and counselling**		**Collaborative care of type 2 diabetes**	
**COM-B component**	**What needs to happen for the target behaviour to occur?**	**Is there a need for change?**	**What needs to happen for the target behaviour to occur?**	**Is there a need for change?**
Physical capability	• Physical skills of health professionals to demonstrate to patients on insulin administration techniques and self-care activities<br>• There is a need for the physical skill of health professionals to provide structured diabetes education	No Change needed	The collaborative care team needs the physical skills to provided collaborative type 2 diabetes care a	No change needed
Psychological capability	• Health professionals and peer diabetes educators need to have the knowledge and skill to provide structured diabetes education and counselling to patients based on patient needs.<br>• Belief, awareness, and perception of the health professionals about diabetes and its complications and treatment	Change needed	• The collaborative care team that includes physicians, nurses, clinical pharmacists, and dietitians or nutritionists need to have the knowledge and skill to provide evidence-based diabetes care in a coordinated way.<br>• There is a need for good communication skills among health professionals, policymakers, and patients in the provision of collaborative care of type 2 diabetes.<br>• Policymakers as part of the diabetes care team need to have the knowledge and skill to lead and coordinate the team.	Change needed

Physical opportunity	• There have to be access to and availability of structured diabetes education and counselling programs to patients at an appropriate space with adequate seats<br>• There is a need for trained and competent multidisciplinary diabetes educators for the provision of structured diabetes education and counselling.<br>• There is a need for updated and appropriate contextualised diabetes education and training materials such as brochures, pamphlets, bulletins, and recorded audio-visuals in a form understandable by all patients.<br>• The structured diabetes education needs to be provided at a convenient time for both patients and health professionals and for a duration of time adequate for the provision of structured diabetes education and counselling.<br>• Availability of continuing medical education (CME) and professional development (CPD) training to health professionals providing diabetes education.	Change needed	• There is a need to establish multidisciplinary collaborative care team that includes nurses, clinical pharmacists, physicians, dietitians or nutritionists, administrative bodies of the hospital, and Ministry of Health of Ethiopia.<br>• There need to be a guideline that guide collaborative work that describes<br>• clear roles, accountabilities, and responsibilities of each member of the collaborative care team.<br>• There should be adequate and trained manpower including dedicated clinical pharmacists and dietitians or nutritionists assigned at the diabetes centre,<br>• There is a need to organise seminar and journal club sessions for the multidisciplinary collaborative care team about how to work collaboratively. There need to be a separate room for clinical pharmacists for the provision of clinical pharmacy services as part of the collaborative care.<br>• There is a need for adequate time and space for the provision of collaborative care of type 2 diabetes<br>• There needs policy support for collaborative care to function at the diabetes centre	Change needed

Social opportunity	Communication and coordination among the multidisciplinary health care team members in providing structured diabetes education and counselling.	Change needed	• There is a need to establish mechanisms to improve communication among health professionals such as regular meetings of the multidisciplinary team, feedback mechanism among the care team, and provision of refreshment services at regular meetings to enhance communication and friendly relationships among the collaborative care team members<br>• There need to be mentorship and supervision by policymakers (administrative bodies of the hospital & Ministry of Health of Ethiopia) to improve awareness of health professionals about collaboration in the care of type 2 diabetes.<br>• There is a need to improve communication with other health professionals, particularly physicians, to improve the attitude and perception of the health professionals towards the involvement and role of clinical pharmacists in patient care.	Change needed
Reflective motivation	• The health professionals have to hold beliefs and perceptions that structured diabetes education and counselling services improve type 2 diabetes care through improving knowledge and awareness of patients about type 2 diabetes and its management.<br>• The health professionals should hold the belief and intend to take CME and CPD training regularly to improve their competency in diabetes education and counselling.	Change needed	• The collaborative care team members need to hold beliefs that collaborative care (involving a multidisciplinary team) of type 2 diabetes improves the quality of type 2 care at the diabetes centre.<br>• The health professionals need to intend to work collaboratively in the provision of type 2 diabetes care	Change needed

	• Health professionals need to have the intention and commitment to providing structured diabetes education and counselling services to patients.			
Automatic motivation	Health professionals have to establish routines and habits to provide structured diabetes education and counselling services,<br>The health professionals have to establish routines of taking CME and CPD training regularly to improve their competency to provide evidence-based diabetes education and counselling services.	Change needed	• The collaborative care team including the policymakers and clinical pharmacists need to establish routines and habits to work collaboratively in the care of type 2 diabetes at the diabetes centre.	Change needed
COM-B components:	psychological capability, physical and social opportunity, reflective and automatic motivation		Psychological capability, physical and social opportunity, reflective and automatic motivation	

Table S4: Linking intervention functions to COM-B components using the Behaviour Change Wheel linking Matrix

	Capability Physical	Capability Psychological	Opportunity Physical	Opportunity Social	Motivation Reflective	Motivation Automatic
**Education**<br>Increasing knowledge or understanding		×			×	
**Persuasion**<br>Using communication to induce positive or negative feelings or stimulate action					×	×
**Incentivisation**<br>Creating expectation of reward					×	×
**Coercion**<br>Creating an expectation of punishment or cost					×	×
**Training**<br>Imparting skills	×	×	×			×
**Restriction**<br>Using rules to reduce the opportunity to engage in the target behaviour (or to increase the target behaviour by reducing the opportunity to engage in competing behaviours			×	×		
**Environmental restructuring**<br>Changing the physical or social context			×	×		×
**Modelling**<br>Providing an example for people to aspire to or imitate				×		×
**Enablement**<br>Increasing means/ reducing barriers to increase capability (beyond education and training) or opportunity (beyond environmental restructuring)	×	×	×	×		×

Note: the × shows the appropriate intervention function for the COM-B component.

Table S5: COM-B diagnosis, intervention functions, policy categories, and behaviour change techniques for the identified target behaviours for intervention

Target behaviour	COM-B component	Candidate intervention function	Does the intervention function meet the APEASE criteria (affordability, practicability, effectiveness/cost-effectiveness, acceptability, side-effects/safety, equity)?	Identified policy categories for the intervention functions based on the APEASE criteria	Identified Behaviour change technique based on the APEASE criteria
Provision of structured diabetes education and counselling	Physical and psychological capability, physical and social opportunity, reflective and automatic motivation	**Education**<br>Increasing knowledge or understanding	Yes	Guidelines<br>Service provision	2.2. Feedback on behaviour<br>2.3. Self-monitoring of behaviour<br>7.1. Prompt/cues
		**Persuasion**<br>Using communication to induce positive or negative feelings or stimulate action	Not applicable in our context		
		**Incentivisation**<br>Creating expectation of reward	Not applicable in our context		
		**Coercion**<br>Creating an expectation of punishment or cost	Not applicable in our context		

		**Training**<br>Imparting skills	Yes	Guidelines<br>Service provision	4.1. Instruction on how to perform the behaviour 6.1. demonstration of the behaviour
		**Restriction**<br>Using rules to reduce the opportunity to engage in the target behaviour (or to increase the target behaviour by reducing the opportunity to engage in competing behaviours	Not applicable in our context		
		**Environmental restructuring**<br>Changing the physical or social context	Yes	Guidelines<br>Environmental/social planning	12.1. Restructuring the physical environment<br>12.2. Restructuring the social environment<br>12.5. Adding objects to the environment<br>7.1. Prompt/ques
		**Modelling**<br>Providing an example for people to aspire to or imitate	Yes	Service provision	6.1. Demonstration of the behaviour
		**Enablement**<br>Increasing means/ reducing barriers to increase capability (beyond education and training) or opportunity (beyond environmental restructuring)	Yes	Guidelines<br>Environmental/social planning<br>Service provision	1.1. Goal setting behavior<br>1.4. Action planning<br>3.1. Social support (unspecified).<br>5.2. Salience of consequences
			Education, training, Environmental restructuring, modelling,	**Identified policy categories:** Guidelines, Environmental/social planning,	**Identified behaviour change techniques:**<br>2.2. Feedback on behaviour<br>2.3. Self-monitoring of behaviour<br>7.1. Prompt/cues<br>5.2. Salience of consequences

			and enablement	Service provision	4.1. Instruction on how to perform the behaviour<br>6.1. demonstration of the behaviour<br>12.1. Restructuring the physical environment<br>12.2. Restructuring the social environment<br>12.5. Adding objects to the environment<br>1.1. Goal setting behaviour<br>1.4. Action planning<br>3.1. Social support (unspecified).
Collaborative care of type 2 diabetes	Psychological capability physical and social opportunity reflective and automatic motivation	**Education**<br>Increasing knowledge or understanding	Yes	Guidelines<br>Regulation<br>Service provision	2.3. Self-monitoring of behaviour<br>7.1. prompt/cues
		**Persuasion**<br>Using communication to induce positive or negative feelings or stimulate action	Not applicable in this context		
		**Incentivisation**<br>Creating expectation of reward	Yes	Service provision	2.2. Feedback on behaviour
		**Coercion**<br>Creating an expectation of punishment or cost	Not acceptable by health professionals		
		**Training**<br>Imparting skills	Yes	Guidelines<br>Regulation<br>Service provision	4.1. Instruction on how to perform the behaviour
		**Restriction**<br>Using rules to reduce the opportunity to engage in the target behaviour (or to	Not applicable in this context	None	None

		increase the target behaviour by reducing the opportunity to engage in competing behaviours			
		**Environmental restructuring**<br>Changing the physical or social context	Yes	Guidelines<br>Regulation<br>Environmental/social planning	12.1. Restructuring the physical environment<br>12.2. Restructuring the social environment<br>12.5. Adding objects to the environment
		**Modelling**<br>Providing an example for people to aspire to or imitate	Yes		6.1. Demonstration of the behaviour
		**Enablement**<br>Increasing means/ reducing barriers to increase capability (beyond education and training) or opportunity (beyond environmental restructuring)	Yes	Guidelines<br>Regulation<br>Environmental/social planning<br>Service provision	1.1. Goal setting behaviour<br>1.4. Action planning<br>3.1. Social support (unspecified).<br>3.2. Social support (practical)<br>1.2. Problem solving
		**Identified intervention functions:**	Education, incentivisation, training, Environmental restructuring, modeling, and enablement	**Identified policy categories**:<br>Guidelines<br>Regulation,<br>Environmental/social planning,<br>Service provision	Identified behavior change techniques:<br>2.3. Self-monitoring of behaviour<br>7.1. prompt/cues<br>2.2. Feedback on behaviour<br>4.1. Instruction on how to perform the behaviour<br>12.1. Restructuring the physical environment<br>12.2. Restructuring the social environment<br>12.5. Adding objects to the environment

					6.1. Demonstration of the behaviour<br>1.1. Goal setting behaviour<br>1.4. Action planning<br>3.1. Social support (unspecified).<br>3.2. Social support (practical)<br>1.2. Problem solving

Table S6: Mode of delivery structured diabetes education

Se. No	Behaviour change technique	Operationalisation of the behaviour change technique
1	2.2. Feedback on behaviour	• Diabetes education checklists to audit diabetes education and counselling is provided or not<br>• Meeting sessions of the diabetes educators to discuss and receive feedback about the structured diabetes education being given to patients<br>• Supervision and mentorship by the policymakers and senior staff on the provision of structured diabetes education and counselling
2	2.3. Self-monitoring of behaviour	• Develop tools to document structured diabetes education and counselling provided (diabetes education and counselling checklist)
3	7.1. Prompt/cues	• Diabetes patient education materials (including manual) that guide structured diabetes education.<br>• Diabetes education and counselling would be guided by the diabetes patient educational materials (diabetes education manual and diabetes education checklist)<br>• Patient resources for the provision of structured diabetes education
4	5.2. Salience of consequences	• Provide video-assisted case notes to diabetes education providers about the consequences of lack of structured diabetes education and counselling
5	4.1. Instruction on how to perform the behaviour	• Train diabetes educators' team of health professionals of various disciplines on how to provide structured diabetes education using illustrations like flow diagram which does what and, how and when the diabetes education should be provided<br>• Provide Diabetes educational materials and manuals for the diabetes educators
6	6.1. demonstration of the behaviour	• Provide practical skills training like role plays and videos and other educational materials assisted training to diabetes on how to provide diabetes education and counselling to the health professionals
7	12.1. Restructuring the physical environment	• Reorganise the diabetes education space convenient to patients and providers<br>• Manuals for the diabetes educators that assist the provision of structured diabetes education and counselling
8	12.2. Restructuring the social environment	• Establish a multidisciplinary diabetes educators team consisting of nurses, clinical pharmacists, physicians, dietitians or nutritionists, peer diabetes educators, and policymakers<br>• The members of the diabetes educators team come together and discuss and agree on the appropriate time and space in the provision for structured diabetes education and counselling
9	12.5. Adding objects to the environment	• Diabetes education materials like brochures, leaflets, audio-visuals; diabetes education checklist; computerised patient referral forms for patients requiring education and counselling; and patient diary to help diabetes educators provide structured diabetes education.

		• Manual for the provision of structured diabetes education and counselling on who does what, when, how often, and with whom
10	1.1. Goal setting behaviour	• Diabetes educators team sets goals to be achieved for provision of diabetes education and counselling<br>• Train and educate the diabetes educator team to acquire the desired competency and behaviour to provide structured diabetes education and training.<br>• Educate, persuade, and enable the diabetes educators to increase their self-confidence in providing structured diabetes education<br>• Continuing diabetes education meetings and workshops
11	1.4. Action planning	• Team of diabetes educators come together to plan on the structure, frequency, duration, and intensity of diabetes education sessions and develop if, then plans.
12	3.1. Social support (unspecified).	• Academic detailing or outreach services
